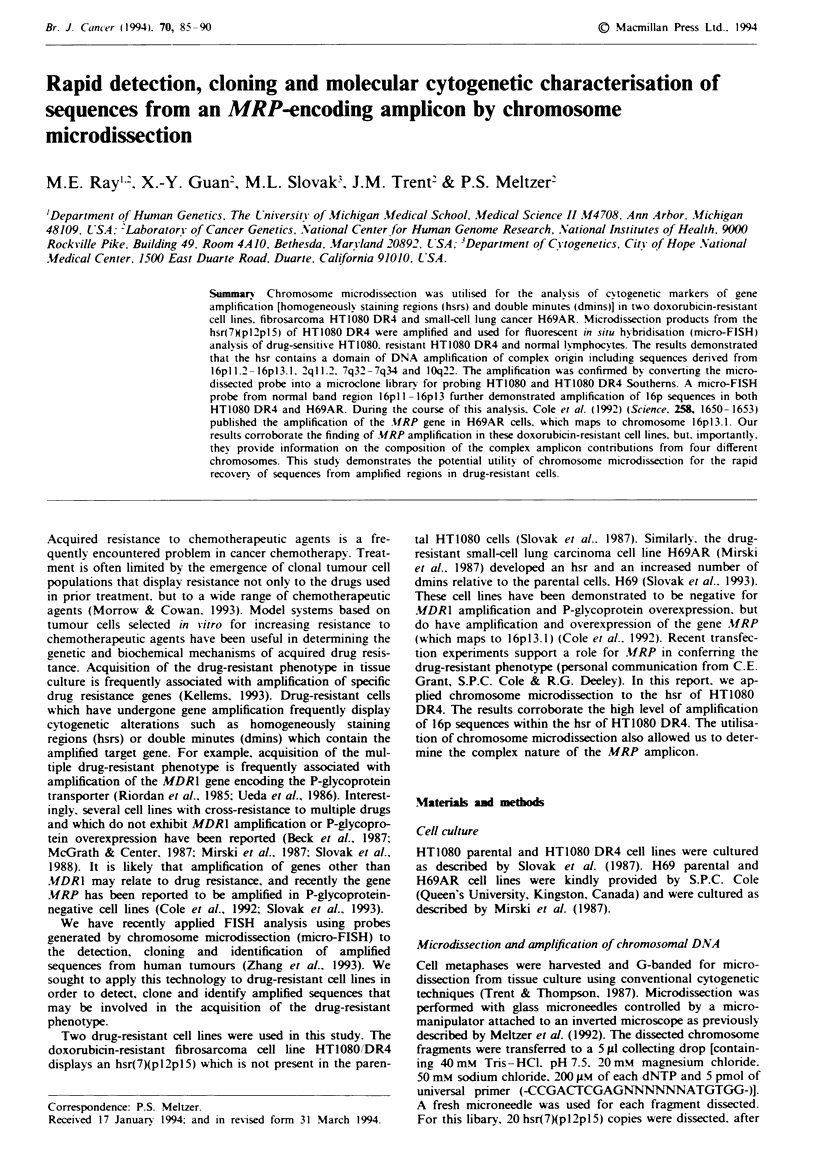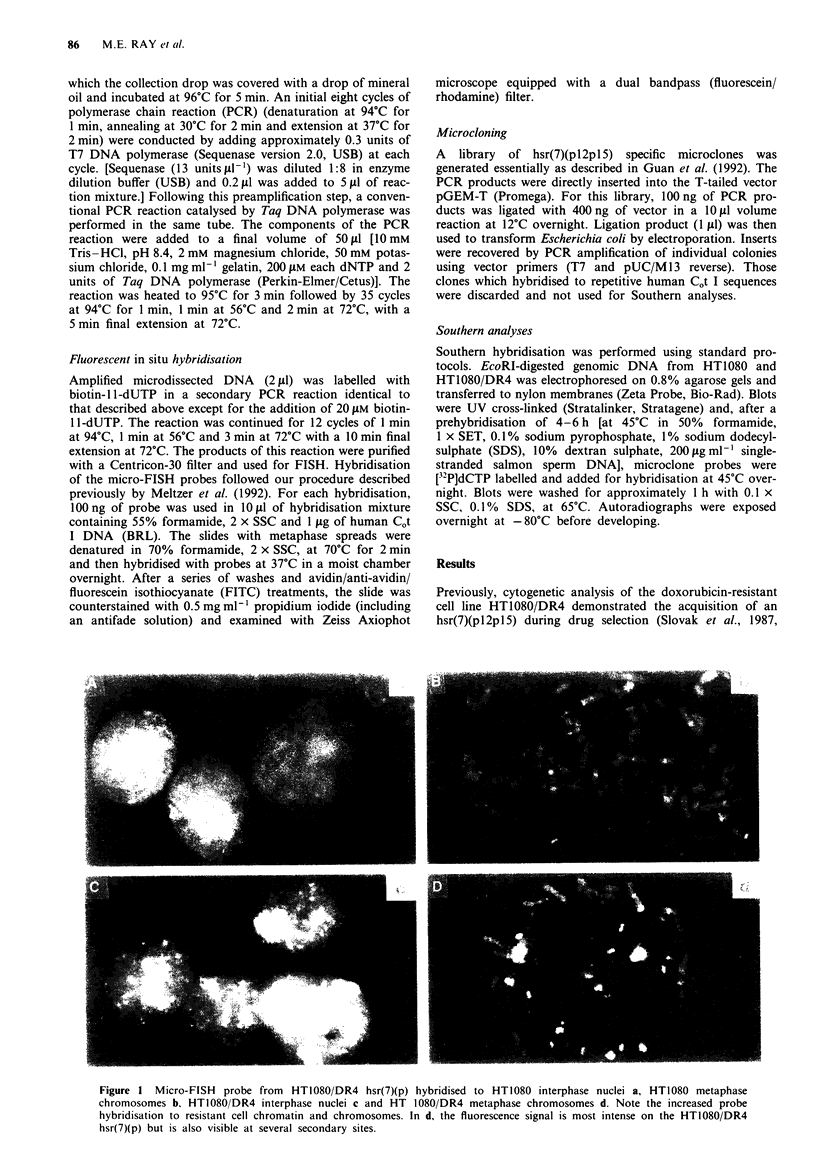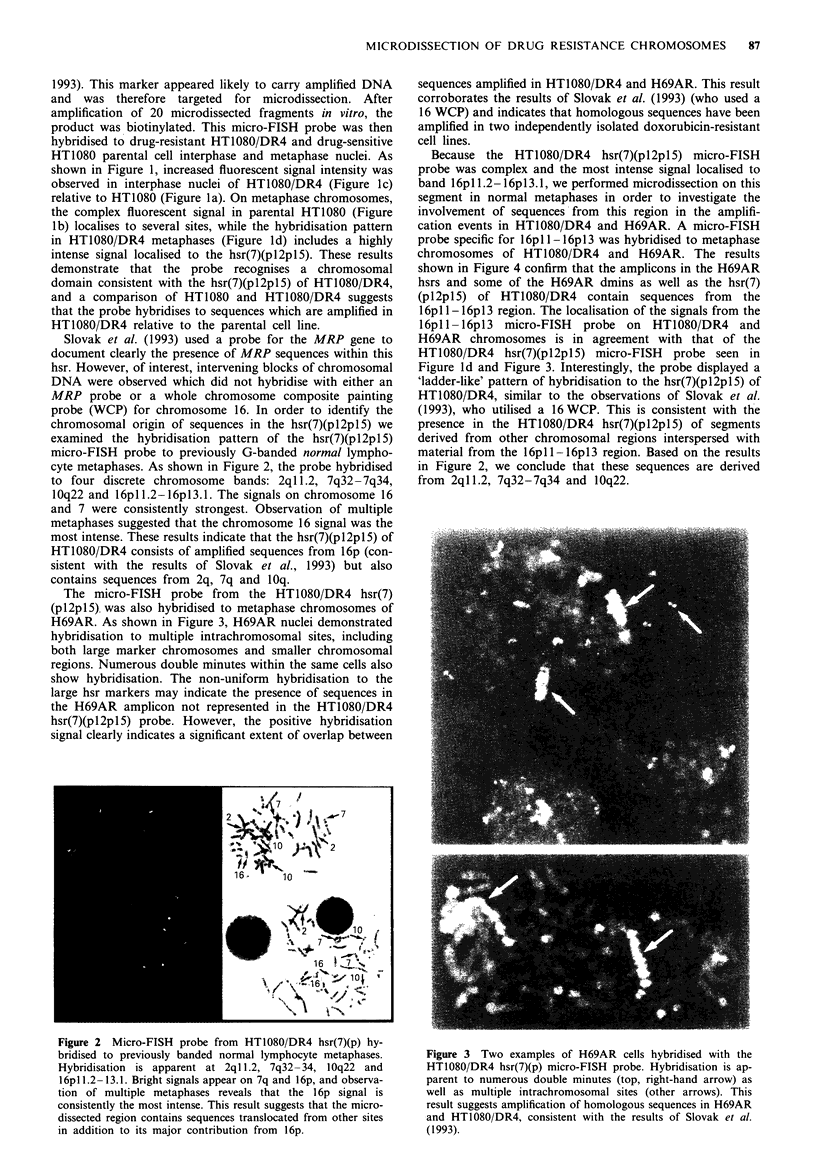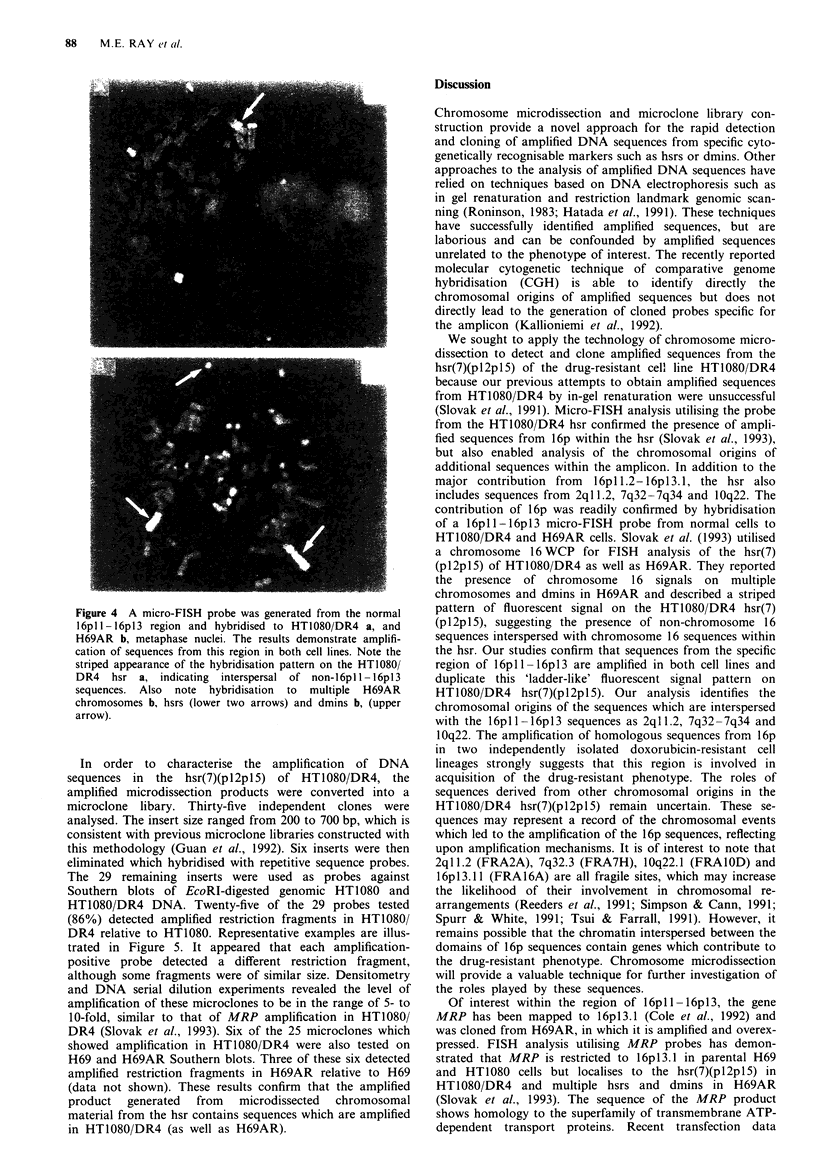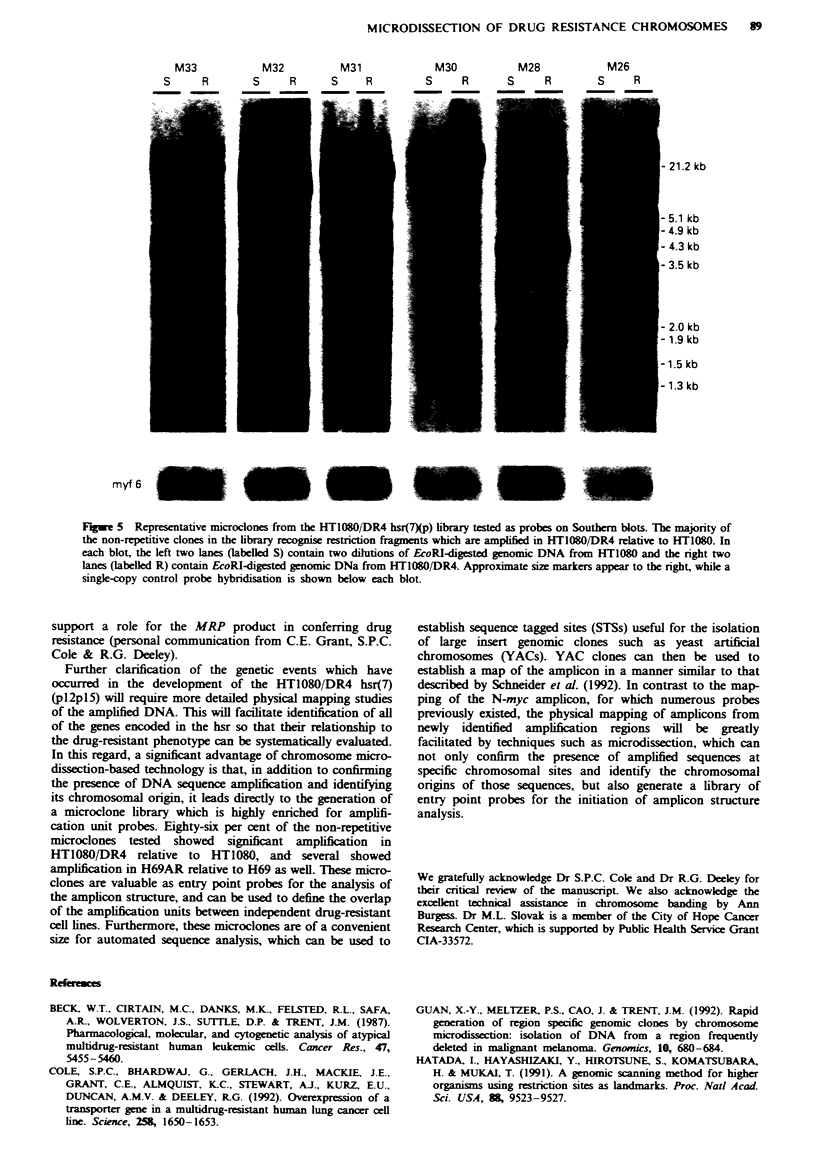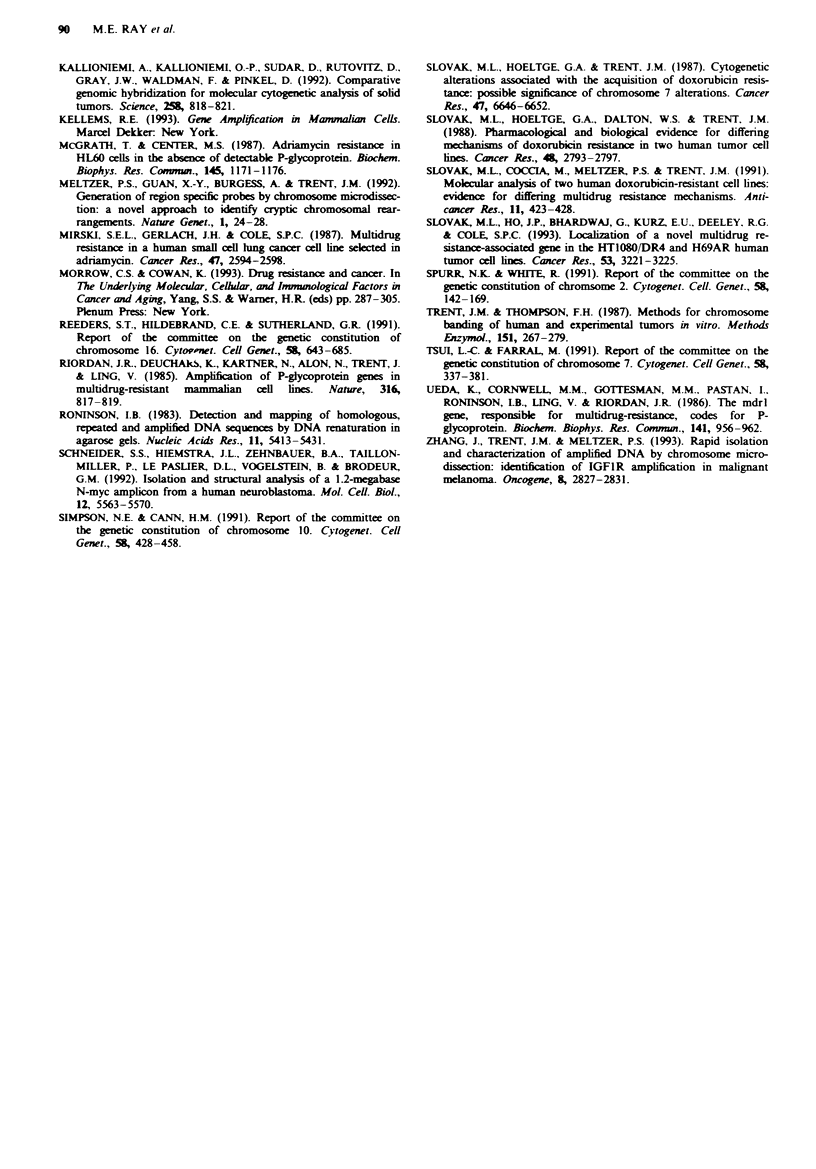# Rapid detection, cloning and molecular cytogenetic characterisation of sequences from an MRP-encoding amplicon by chromosome microdissection.

**DOI:** 10.1038/bjc.1994.254

**Published:** 1994-07

**Authors:** M. E. Ray, X. Y. Guan, M. L. Slovak, J. M. Trent, P. S. Meltzer

**Affiliations:** Department of Human Genetics, University of Michigan Medical School, Ann Arbor 48109.

## Abstract

**Images:**


Br. J. Cancer (1994), 70, 85-90 © Macmillan Press Ltd., 1994

### Rapid detection, cloning and molecular cytogenetic characterisation of sequences from an *MRP*-encoding amplicon by chromosome microdissection

M.E. Ray[1,2], X.-Y. Guan[2], M.L. Slovak[3], J.M. Trent[2] & P.S. Meltzer[2]

[1]*Department of Human Genetics, The University of Michigan Medical School, Medical Science II M4708, Ann Arbor, Michigan 48109, USA;* [2]*Laboratory of Cancer Genetics, National Center for Human Genome Research, National Institutes of Health, 9000 Rockville Pike, Building 49, Room 4A10, Bethesda, Maryland 20892, USA;* [3]*Department of Cytogenetics, City of Hope National Medical Center, 1500 East Duarte Road, Duarte, California 91010, USA.*

**Summary** Chromosome microdissection was utilised for the analysis of cytogenetic markers of gene amplification [homogeneously staining regions (hsrs) and double minutes (dmins)] in two doxorubicin-resistant cell lines, fibrosarcoma HT1080 DR4 and small-cell lung cancer H69AR. Microdissection products from the hsr(7)(p12p15) of HT1080 DR4 were amplified and used for fluorescent *in situ* hybridisation (micro-FISH) analysis of drug-sensitive HT1080, resistant HT1080 DR4 and normal lymphocytes. The results demonstrated that the hsr contains a domain of DNA amplification of complex origin including sequences derived from 16p11.2-16p13.1, 2q11.2, 7q32-7q34 and 10q22. The amplification was confirmed by converting the microdissected probe into a microclone library for probing HT1080 and HT1080 DR4 Southerns. A micro-FISH probe from normal band region 16p11-16p13 further demonstrated amplification of 16p sequences in both HT1080 DR4 and H69AR. During the course of this analysis, Cole *et al.* (1992) (*Science*, **258**, 1650-1653) published the amplification of the *MRP* gene in H69AR cells, which maps to chromosome 16p13.1. Our results corroborate the finding of *MRP* amplification in these doxorubicin-resistant cell lines, but, importantly, they provide information on the composition of the complex amplicon contributions from four different chromosomes. This study demonstrates the potential utility of chromosome microdissection for the rapid recovery of sequences from amplified regions in drug-resistant cells.

Acquired resistance to chemotherapeutic agents is a frequently encountered problem in cancer chemotherapy. Treatment is often limited by the emergence of clonal tumour cell populations that display resistance not only to the drugs used in prior treatment, but to a wide range of chemotherapeutic agents (Morrow & Cowan, 1993). Model systems based on tumour cells selected *in vitro* for increasing resistance to chemotherapeutic agents have been useful in determining the genetic and biochemical mechanisms of acquired drug resistance. Acquisition of the drug-resistant phenotype in tissue culture is frequently associated with amplification of specific drug resistance genes (Kellems, 1993). Drug-resistant cells which have undergone gene amplification frequently display cytogenetic alterations such as homogeneously staining regions (hsrs) or double minutes (dmins) which contain the amplified target gene. For example, acquisition of the multiple drug-resistant phenotype is frequently associated with amplification of the *MDR*1 gene encoding the P-glycoprotein transporter (Riordan *et al.*, 1985; Ueda *et al.*, 1986). Interestingly, several cell lines with cross-resistance to multiple drugs and which do not exhibit *MDR*1 amplification or P-glycoprotein overexpression have been reported (Beck *et al.*, 1987; McGrath & Center, 1987; Mirski *et al.*, 1987; Slovak *et al.*, 1988). It is likely that amplification of genes other than *MDR*1 may relate to drug resistance, and recently the gene *MRP* has been reported to be amplified in P-glycoprotein-negative cell lines (Cole *et al.*, 1992; Slovak *et al.*, 1993).

We have recently applied FISH analysis using probes generated by chromosome microdissection (micro-FISH) to the detection, cloning and identification of amplified sequences from human tumours (Zhang *et al.*, 1993). We sought to apply this technology to drug-resistant cell lines in order to detect, clone and identify amplified sequences that may be involved in the acquisition of the drug-resistant phenotype.

Two drug-resistant cell lines were used in this study. The doxorubicin-resistant fibrosarcoma cell line HT1080/DR4 displays an hsr(7)(p12p15) which is not present in the parental HT1080 cells (Slovak *et al.*, 1987). Similarly, the drug-resistant small-cell lung carcinoma cell line H69AR (Mirski *et al.*, 1987) developed an hsr and an increased number of dmins relative to the parental cells, H69 (Slovak *et al.*, 1993). These cell lines have been demonstrated to be negative for *MDR*1 amplification and P-glycoprotein overexpression, but do have amplification and overexpression of the gene *MRP* (which maps to 16p13.1) (Cole *et al.*, 1992). Recent transfection experiments support a role for *MRP* in conferring the drug-resistant phenotype (personal communication from C.E. Grant, S.P.C. Cole & R.G. Deeley). In this report, we applied chromosome microdissection to the hsr of HT1080 DR4. The results corroborate the high level of amplification of 16p sequences within the hsr of HT1080/DR4. The utilisation of chromosome microdissection also allowed us to determine the complex nature of the *MRP* amplicon.

### Materials and methods

### Cell culture

HT1080 parental and HT1080/DR4 cell lines were cultured as described by Slovak *et al.* (1987). H69 parental and H69AR cell lines were kindly provided by S.P.C. Cole (Queen's University, Kingston, Canada) and were cultured as described by Mirski *et al.* (1987).

### Microdissection and amplification of chromosomal DNA

Cell metaphases were harvested and G-banded for microdissection from tissue culture using conventional cytogenetic techniques (Trent & Thompson, 1987). Microdissection was performed with glass microneedles controlled by a micromanipulator attached to an inverted microscope as previously described by Meltzer *et al.* (1992). The dissected chromosome fragments were transferred to a 5 μl collecting drop [containing 40 mM Tris-HCl, pH 7.5, 20 mM magnesium chloride, 50 mM sodium chloride, 200 μM of each dNTP and 5 pmol of universal primer (-CCGACTCGAGNNNNNNATGTGG-)]. A fresh microneedle was used for each fragment dissected. For this libary, 20 hsr(7)(p12p15) copies were dissected, after

Correspondence: P.S. Meltzer.
Received 17 January 1994; and in revised form 31 March 1994.

which the collection drop was covered with a drop of mineral oil and incubated at 96°C for 5 min. An initial eight cycles of polymerase chain reaction (PCR) (denaturation at 94°C for 1 min, annealing at 30°C for 2 min and extension at 37°C for 2 min) were conducted by adding approximately 0.3 units of T7 DNA polymerase (Sequenase version 2.0, USB) at each cycle. [Sequenase (13 units $\mu l^{-1}$) was diluted 1:8 in enzyme dilution buffer (USB) and 0.2 µl was added to 5 µl of reaction mixture.] Following this preamplification step, a conventional PCR reaction catalysed by *Taq* DNA polymerase was performed in the same tube. The components of the PCR reaction were added to a final volume of 50 µl [10 mM Tris-HCl, pH 8.4, 2 mM magnesium chloride, 50 mM potassium chloride, 0.1 mg $ml^{-1}$ gelatin, 200 µM each dNTP and 2 units of *Taq* DNA polymerase (Perkin-Elmer/Cetus)]. The reaction was heated to 95°C for 3 min followed by 35 cycles at 94°C for 1 min, 1 min at 56°C and 2 min at 72°C, with a 5 min final extension at 72°C.

### *Fluorescent* in situ *hybridisation*

Amplified microdissected DNA (2 µl) was labelled with biotin-11-dUTP in a secondary PCR reaction identical to that described above except for the addition of 20 µM biotin-11-dUTP. The reaction was continued for 12 cycles of 1 min at 94°C, 1 min at 56°C and 3 min at 72°C with a 10 min final extension at 72°C. The products of this reaction were purified with a Centricon-30 filter and used for FISH. Hybridisation of the micro-FISH probes followed our procedure described previously by Meltzer *et al.* (1992). For each hybridisation, 100 ng of probe was used in 10 µl of hybridisation mixture containing 55% formamide, 2 × SSC and 1 µg of human $C_0t$ I DNA (BRL). The slides with metaphase spreads were denatured in 70% formamide, 2 × SSC, at 70°C for 2 min and then hybridised with probes at 37°C in a moist chamber overnight. After a series of washes and avidin/anti-avidin/fluorescein isothiocyanate (FITC) treatments, the slide was counterstained with 0.5 mg $ml^{-1}$ propidium iodide (including an antifade solution) and examined with Zeiss Axiophot microscope equipped with a dual bandpass (fluorescein/rhodamine) filter.

### *Microcloning*

A library of hsr(7)(p12p15) specific microclones was generated essentially as described in Guan *et al.* (1992). The PCR products were directly inserted into the T-tailed vector pGEM-T (Promega). For this library, 100 ng of PCR products was ligated with 400 ng of vector in a 10 µl volume reaction at 12°C overnight. Ligation product (1 µl) was then used to transform *Escherichia coli* by electroporation. Inserts were recovered by PCR amplification of individual colonies using vector primers (T7 and pUC/M13 reverse). Those clones which hybridised to repetitive human $C_0t$ I sequences were discarded and not used for Southern analyses.

### *Southern analyses*

Southern hybridisation was performed using standard protocols. *Eco*RI-digested genomic DNA from HT1080 and HT1080/DR4 was electrophoresed on 0.8% agarose gels and transferred to nylon membranes (Zeta Probe, Bio-Rad). Blots were UV cross-linked (Stratalinker, Stratagene) and, after a prehybridisation of 4-6 h [at 45°C in 50% formamide, 1 × SET, 0.1% sodium pyrophosphate, 1% sodium dodecylsulphate (SDS), 10% dextran sulphate, 200 µg $ml^{-1}$ single-stranded salmon sperm DNA], microclone probes were [$^{32}P$]dCTP labelled and added for hybridisation at 45°C overnight. Blots were washed for approximately 1 h with 0.1 × SSC, 0.1% SDS, at 65°C. Autoradiographs were exposed overnight at −80°C before developing.

### Results

Previously, cytogenetic analysis of the doxorubicin-resistant cell line HT1080/DR4 demonstrated the acquisition of an hsr(7)(p12p15) during drug selection (Slovak *et al.*, 1987,

**Figure 1** Micro-FISH probe from HT1080/DR4 hsr(7)(p) hybridised to HT1080 interphase nuclei **a**, HT1080 metaphase chromosomes **b**, HT1080/DR4 interphase nuclei **c** and HT 1080/DR4 metaphase chromosomes **d**. Note the increased probe hybridisation to resistant cell chromatin and chromosomes. In **d**, the fluorescence signal is most intense on the HT1080/DR4 hsr(7)(p) but is also visible at several secondary sites.

1993). This marker appeared likely to carry amplified DNA and was therefore targeted for microdissection. After amplification of 20 microdissected fragments *in vitro*, the product was biotinylated. This micro-FISH probe was then hybridised to drug-resistant HT1080/DR4 and drug-sensitive HT1080 parental cell interphase and metaphase nuclei. As shown in Figure 1, increased fluorescent signal intensity was observed in interphase nuclei of HT1080/DR4 (Figure 1c) relative to HT1080 (Figure 1a). On metaphase chromosomes, the complex fluorescent signal in parental HT1080 (Figure 1b) localises to several sites, while the hybridisation pattern in HT1080/DR4 metaphases (Figure 1d) includes a highly intense signal localised to the hsr(7)(p12p15). These results demonstrate that the probe recognises a chromosomal domain consistent with the hsr(7)(p12p15) of HT1080/DR4, and a comparison of HT1080 and HT1080/DR4 suggests that the probe hybridises to sequences which are amplified in HT1080/DR4 relative to the parental cell line.

Slovak *et al.* (1993) used a probe for the *MRP* gene to document clearly the presence of *MRP* sequences within this hsr. However, of interest, intervening blocks of chromosomal DNA were observed which did not hybridise with either an *MRP* probe or a whole chromosome composite painting probe (WCP) for chromosome 16. In order to identify the chromosomal origin of sequences in the hsr(7)(p12p15) we examined the hybridisation pattern of the hsr(7)(p12p15) micro-FISH probe to previously G-banded *normal* lymphocyte metaphases. As shown in Figure 2, the probe hybridised to four discrete chromosome bands: 2q11.2, 7q32–7q34, 10q22 and 16p11.2–16p13.1. The signals on chromosome 16 and 7 were consistently strongest. Observation of multiple metaphases suggested that the chromosome 16 signal was the most intense. These results indicate that the hsr(7)(p12p15) of HT1080/DR4 consists of amplified sequences from 16p (consistent with the results of Slovak *et al.*, 1993) but also contains sequences from 2q, 7q and 10q.

The micro-FISH probe from the HT1080/DR4 hsr(7)(p12p15) was also hybridised to metaphase chromosomes of H69AR. As shown in Figure 3, H69AR nuclei demonstrated hybridisation to multiple intrachromosomal sites, including both large marker chromosomes and smaller chromosomal regions. Numerous double minutes within the same cells also show hybridisation. The non-uniform hybridisation to the large hsr markers may indicate the presence of sequences in the H69AR amplicon not represented in the HT1080/DR4 hsr(7)(p12p15) probe. However, the positive hybridisation signal clearly indicates a significant extent of overlap between sequences amplified in HT1080/DR4 and H69AR. This result corroborates the results of Slovak *et al.* (1993) (who used a 16 WCP) and indicates that homologous sequences have been amplified in two independently isolated doxorubicin-resistant cell lines.

Because the HT1080/DR4 hsr(7)(p12p15) micro-FISH probe was complex and the most intense signal localised to band 16p11.2–16p13.1, we performed microdissection on this segment in normal metaphases in order to investigate the involvement of sequences from this region in the amplification events in HT1080/DR4 and H69AR. A micro-FISH probe specific for 16p11–16p13 was hybridised to metaphase chromosomes of HT1080/DR4 and H69AR. The results shown in Figure 4 confirm that the amplicons in the H69AR hsrs and some of the H69AR dmins as well as the hsr(7)(p12p15) of HT1080/DR4 contain sequences from the 16p11–16p13 region. The localisation of the signals from the 16p11–16p13 micro-FISH probe on HT1080/DR4 and H69AR chromosomes is in agreement with that of the HT1080/DR4 hsr(7)(p12p15) micro-FISH probe seen in Figure 1d and Figure 3. Interestingly, the probe displayed a 'ladder-like' pattern of hybridisation to the hsr(7)(p12p15) of HT1080/DR4, similar to the observations of Slovak *et al.* (1993), who utilised a 16 WCP. This is consistent with the presence in the HT1080/DR4 hsr(7)(p12p15) of segments derived from other chromosomal regions interspersed with material from the 16p11–16p13 region. Based on the results in Figure 2, we conclude that these sequences are derived from 2q11.2, 7q32–7q34 and 10q22.

**Figure 2** Micro-FISH probe from HT1080/DR4 hsr(7)(p) hybridised to previously banded normal lymphocyte metaphases. Hybridisation is apparent at 2q11.2, 7q32–34, 10q22 and 16p11.2–13.1. Bright signals appear on 7q and 16p, and observation of multiple metaphases reveals that the 16p signal is consistently the most intense. This result suggests that the microdissected region contains sequences translocated from other sites in addition to its major contribution from 16p.

**Figure 3** Two examples of H69AR cells hybridised with the HT1080/DR4 hsr(7)(p) micro-FISH probe. Hybridisation is apparent to numerous double minutes (top, right-hand arrow) as well as multiple intrachromosomal sites (other arrows). This result suggests amplification of homologous sequences in H69AR and HT1080/DR4, consistent with the results of Slovak *et al.* (1993).

**Figure 4** A micro-FISH probe was generated from the normal 16p11-16p13 region and hybridised to HT1080/DR4 **a**, and H69AR **b**, metaphase nuclei. The results demonstrate amplification of sequences from this region in both cell lines. Note the striped appearance of the hybridisation pattern on the HT1080/DR4 hsr **a**, indicating interspersal of non-16p11-16p13 sequences. Also note hybridisation to multiple H69AR chromosomes **b**, hsrs (lower two arrows) and dmins **b**, (upper arrow).

In order to characterise the amplification of DNA sequences in the hsr(7)(p12p15) of HT1080/DR4, the amplified microdissection products were converted into a microclone libary. Thirty-five independent clones were analysed. The insert size ranged from 200 to 700 bp, which is consistent with previous microclone libraries constructed with this methodology (Guan *et al.*, 1992). Six inserts were then eliminated which hybridised with repetitive sequence probes. The 29 remaining inserts were used as probes against Southern blots of *Eco*RI-digested genomic HT1080 and HT1080/DR4 DNA. Twenty-five of the 29 probes tested (86%) detected amplified restriction fragments in HT1080/DR4 relative to HT1080. Representative examples are illustrated in Figure 5. It appeared that each amplification-positive probe detected a different restriction fragment, although some fragments were of similar size. Densitometry and DNA serial dilution experiments revealed the level of amplification of these microclones to be in the range of 5- to 10-fold, similar to that of *MRP* amplification in HT1080/DR4 (Slovak *et al.*, 1993). Six of the 25 microclones which showed amplification in HT1080/DR4 were also tested on H69 and H69AR Southern blots. Three of these six detected amplified restriction fragments in H69AR relative to H69 (data not shown). These results confirm that the amplified product generated from microdissected chromosomal material from the hsr contains sequences which are amplified in HT1080/DR4 (as well as H69AR).

### Discussion

Chromosome microdissection and microclone library construction provide a novel approach for the rapid detection and cloning of amplified DNA sequences from specific cytogenetically recognisable markers such as hsrs or dmins. Other approaches to the analysis of amplified DNA sequences have relied on techniques based on DNA electrophoresis such as in gel renaturation and restriction landmark genomic scanning (Roninson, 1983; Hatada *et al.*, 1991). These techniques have successfully identified amplified sequences, but are laborious and can be confounded by amplified sequences unrelated to the phenotype of interest. The recently reported molecular cytogenetic technique of comparative genome hybridisation (CGH) is able to identify directly the chromosomal origins of amplified sequences but does not directly lead to the generation of cloned probes specific for the amplicon (Kallioniemi *et al.*, 1992).

We sought to apply the technology of chromosome microdissection to detect and clone amplified sequences from the hsr(7)(p12p15) of the drug-resistant cell line HT1080/DR4 because our previous attempts to obtain amplified sequences from HT1080/DR4 by in-gel renaturation were unsuccessful (Slovak *et al.*, 1991). Micro-FISH analysis utilising the probe from the HT1080/DR4 hsr confirmed the presence of amplified sequences from 16p within the hsr (Slovak *et al.*, 1993), but also enabled analysis of the chromosomal origins of additional sequences within the amplicon. In addition to the major contribution from 16p11.2-16p13.1, the hsr also includes sequences from 2q11.2, 7q32-7q34 and 10q22. The contribution of 16p was readily confirmed by hybridisation of a 16p11-16p13 micro-FISH probe from normal cells to HT1080/DR4 and H69AR cells. Slovak *et al.* (1993) utilised a chromosome 16 WCP for FISH analysis of the hsr(7)(p12p15) of HT1080/DR4 as well as H69AR. They reported the presence of chromosome 16 signals on multiple chromosomes and dmins in H69AR and described a striped pattern of fluorescent signal on the HT1080/DR4 hsr(7)(p12p15), suggesting the presence of non-chromosome 16 sequences interspersed with chromosome 16 sequences within the hsr. Our studies confirm that sequences from the specific region of 16p11-16p13 are amplified in both cell lines and duplicate this 'ladder-like' fluorescent signal pattern on HT1080/DR4 hsr(7)(p12p15). Our analysis identifies the chromosomal origins of the sequences which are interspersed with the 16p11-16p13 sequences as 2q11.2, 7q32-7q34 and 10q22. The amplification of homologous sequences from 16p in two independently isolated doxorubicin-resistant cell lineages strongly suggests that this region is involved in acquisition of the drug-resistant phenotype. The roles of sequences derived from other chromosomal origins in the HT1080/DR4 hsr(7)(p12p15) remain uncertain. These sequences may represent a record of the chromosomal events which led to the amplification of the 16p sequences, reflecting upon amplification mechanisms. It is of interest to note that 2q11.2 (FRA2A), 7q32.3 (FRA7H), 10q22.1 (FRA10D) and 16p13.11 (FRA16A) are all fragile sites, which may increase the likelihood of their involvement in chromosomal rearrangements (Reeders *et al.*, 1991; Simpson & Cann, 1991; Spurr & White, 1991; Tsui & Farrall, 1991). However, it remains possible that the chromatin interspersed between the domains of 16p sequences contain genes which contribute to the drug-resistant phenotype. Chromosome microdissection will provide a valuable technique for further investigation of the roles played by these sequences.

Of interest within the region of 16p11-16p13, the gene *MRP* has been mapped to 16p13.1 (Cole *et al.*, 1992) and was cloned from H69AR, in which it is amplified and overexpressed. FISH analysis utilising *MRP* probes has demonstrated that *MRP* is restricted to 16p13.1 in parental H69 and HT1080 cells but localises to the hsr(7)(p12p15) in HT1080/DR4 and multiple hsrs and dmins in H69AR (Slovak *et al.*, 1993). The sequence of the *MRP* product shows homology to the superfamily of transmembrane ATP-dependent transport proteins. Recent transfection data

**Figure 5** Representative microclones from the HT1080/DR4 hsr(7)(p) library tested as probes on Southern blots. The majority of the non-repetitive clones in the library recognise restriction fragments which are amplified in HT1080/DR4 relative to HT1080. In each blot, the left two lanes (labelled S) contain two dilutions of *Eco*RI-digested genomic DNA from HT1080 and the right two lanes (labelled R) contain *Eco*RI-digested genomic DNa from HT1080/DR4. Approximate size markers appear to the right, while a single-copy control probe hybridisation is shown below each blot.

support a role for the *MRP* product in conferring drug resistance (personal communication from C.E. Grant, S.P.C. Cole & R.G. Deeley).

Further clarification of the genetic events which have occurred in the development of the HT1080/DR4 hsr(7) (p12p15) will require more detailed physical mapping studies of the amplified DNA. This will facilitate identification of all of the genes encoded in the hsr so that their relationship to the drug-resistant phenotype can be systematically evaluated. In this regard, a significant advantage of chromosome microdissection-based technology is that, in addition to confirming the presence of DNA sequence amplification and identifying its chromosomal origin, it leads directly to the generation of a microclone library which is highly enriched for amplification unit probes. Eighty-six per cent of the non-repetitive microclones tested showed significant amplification in HT1080/DR4 relative to HT1080, and several showed amplification in H69AR relative to H69 as well. These microclones are valuable as entry point probes for the analysis of the amplicon structure, and can be used to define the overlap of the amplification units between independent drug-resistant cell lines. Furthermore, these microclones are of a convenient size for automated sequence analysis, which can be used to establish sequence tagged sites (STSs) useful for the isolation of large insert genomic clones such as yeast artificial chromosomes (YACs). YAC clones can then be used to establish a map of the amplicon in a manner similar to that described by Schneider *et al.* (1992). In contrast to the mapping of the N-*myc* amplicon, for which numerous probes previously existed, the physical mapping of amplicons from newly identified amplification regions will be greatly facilitated by techniques such as microdissection, which can not only confirm the presence of amplified sequences at specific chromosomal sites and identify the chromosomal origins of those sequences, but also generate a library of entry point probes for the initiation of amplicon structure analysis.

We gratefully acknowledge Dr S.P.C. Cole and Dr R.G. Deeley for their critical review of the manuscript. We also acknowledge the excellent technical assistance in chromosome banding by Ann Burgess. Dr M.L. Slovak is a member of the City of Hope Cancer Research Center, which is supported by Public Health Service Grant CIA-33572.

**References**

BECK, W.T., CIRTAIN, M.C., DANKS, M.K., FELSTED, R.L., SAFA, A.R., WOLVERTON, J.S., SUTTLE, D.P. & TRENT, J.M. (1987). Pharmacological, molecular, and cytogenetic analysis of atypical multidrug-resistant human leukemic cells. *Cancer Res.*, **47**, 5455–5460.

COLE, S.P.C., BHARDWAJ, G., GERLACH, J.H., MACKIE, J.E., GRANT, C.E., ALMQUIST, K.C., STEWART, A.J., KURZ, E.U., DUNCAN, A.M.V. & DEELEY, R.G. (1992). Overexpression of a transporter gene in a multidrug-resistant human lung cancer cell line. *Science*, **258**, 1650–1653.

GUAN, X.-Y., MELTZER, P.S., CAO, J. & TRENT, J.M. (1992). Rapid generation of region specific genomic clones by chromosome microdissection: isolation of DNA from a region frequently deleted in malignant melanoma. *Genomics*, **10**, 680–684.

HATADA, I., HAYASHIZAKI, Y., HIROTSUNE, S., KOMATSUBARA, H. & MUKAI, T. (1991). A genomic scanning method for higher organisms using restriction sites as landmarks. *Proc. Natl Acad. Sci. USA*, **88**, 9523–9527.

KALLIONIEMI, A., KALLIONIEMI, O.-P., SUDAR, D., RUTOVITZ, D., GRAY, J.W., WALDMAN, F. & PINKEL, D. (1992). Comparative genomic hybridization for molecular cytogenetic analysis of solid tumors. *Science*, **258**, 818–821.

KELLEMS, R.E. (1993). *Gene Amplification in Mammalian Cells*. Marcel Dekker: New York.

MCGRATH, T. & CENTER, M.S. (1987). Adriamycin resistance in HL60 cells in the absence of detectable P-glycoprotein. *Biochem. Biophys. Res. Commun.*, **145**, 1171–1176.

MELTZER, P.S., GUAN, X.-Y., BURGESS, A. & TRENT, J.M. (1992). Generation of region specific probes by chromosome microdissection: a novel approach to identify cryptic chromosomal rearrangements. *Nature Genet.*, **1**, 24–28.

MIRSKI, S.E.L., GERLACH, J.H. & COLE, S.P.C. (1987). Multidrug resistance in a human small cell lung cancer cell line selected in adriamycin. *Cancer Res.*, **47**, 2594–2598.

MORROW, C.S. & COWAN, K. (1993). Drug resistance and cancer. In *The Underlying Molecular, Cellular, and Immunological Factors in Cancer and Aging*, Yang, S.S. & Warner, H.R. (eds) pp. 287–305. Plenum Press: New York.

REEDERS, S.T., HILDEBRAND, C.E. & SUTHERLAND, G.R. (1991). Report of the committee on the genetic constitution of chromosome 16. *Cytogenet. Cell Genet.*, **58**, 643–685.

RIORDAN, J.R., DEUCHARS, K., KARTNER, N., ALON, N., TRENT, J. & LING, V. (1985). Amplification of P-glycoprotein genes in multidrug-resistant mammalian cell lines. *Nature*, **316**, 817–819.

RONINSON, I.B. (1983). Detection and mapping of homologous, repeated and amplified DNA sequences by DNA renaturation in agarose gels. *Nucleic Acids Res.*, **11**, 5413–5431.

SCHNEIDER, S.S., HIEMSTRA, J.L., ZEHNBAUER, B.A., TAILLON-MILLER, P., LE PASLIER, D.L., VOGELSTEIN, B. & BRODEUR, G.M. (1992). Isolation and structural analysis of a 1.2-megabase N-myc amplicon from a human neuroblastoma. *Mol. Cell. Biol.*, **12**, 5563–5570.

SIMPSON, N.E. & CANN, H.M. (1991). Report of the committee on the genetic constitution of chromosome 10. *Cytogenet. Cell Genet.*, **58**, 428–458.

SLOVAK, M.L., HOELTGE, G.A. & TRENT, J.M. (1987). Cytogenetic alterations associated with the acquisition of doxorubicin resistance: possible significance of chromosome 7 alterations. *Cancer Res.*, **47**, 6646–6652.

SLOVAK, M.L., HOELTGE, G.A., DALTON, W.S. & TRENT, J.M. (1988). Pharmacological and biological evidence for differing mechanisms of doxorubicin resistance in two human tumor cell lines. *Cancer Res.*, **48**, 2793–2797.

SLOVAK, M.L., COCCIA, M., MELTZER, P.S. & TRENT, J.M. (1991). Molecular analysis of two human doxorubicin-resistant cell lines: evidence for differing multidrug resistance mechanisms. *Anticancer Res.*, **11**, 423–428.

SLOVAK, M.L., HO, J.P., BHARDWAJ, G., KURZ, E.U., DEELEY, R.G. & COLE, S.P.C. (1993). Localization of a novel multidrug resistance-associated gene in the HT1080/DR4 and H69AR human tumor cell lines. *Cancer Res.*, **53**, 3221–3225.

SPURR, N.K. & WHITE, R. (1991). Report of the committee on the genetic constitution of chromsome 2. *Cytogenet. Cell. Genet.*, **58**, 142–169.

TRENT, J.M. & THOMPSON, F.H. (1987). Methods for chromosome banding of human and experimental tumors *in vitro*. *Methods Enzymol.*, **151**, 267–279.

TSUI, L.-C. & FARRAL, M. (1991). Report of the committee on the genetic constitution of chromosome 7. *Cytogenet. Cell Genet.*, **58**, 337–381.

UEDA, K., CORNWELL, M.M., GOTTESMAN, M.M., PASTAN, I., RONINSON, I.B., LING, V. & RIORDAN, J.R. (1986). The mdr1 gene, responsible for multidrug-resistance, codes for P-glycoprotein. *Biochem. Biophys. Res. Commun.*, **141**, 956–962.

ZHANG, J., TRENT, J.M. & MELTZER, P.S. (1993). Rapid isolation and characterization of amplified DNA by chromosome microdissection: identification of IGF1R amplification in malignant melanoma. *Oncogene*, **8**, 2827–2831.